# Boredom Proneness Predicts Self-Assessed Decision Errors in Sports but Is Unrelated to Risk Taking in General

**DOI:** 10.3390/ijerph19063479

**Published:** 2022-03-15

**Authors:** Wanja Wolff, Maik Bieleke, Lucas Keller

**Affiliations:** 1 Department of Sport Science, University of Konstanz, 78457 Konstanz, Germany; wanja.wolff@uni-konstanz.de (W.W.); maik.bieleke@uni-konstanz.de (M.B.); 2 Institute of Educational Science, Educational Psychology, University of Bern, 3012 Bern, Switzerland; 3 Department of Psychology, University of Konstanz, 78457 Konstanz, Germany

**Keywords:** risk taking, extreme sports, esports, boredom, decision making

## Abstract

**Introduction**: Boredom proneness is linked to poor self-regulation, leading to poor decision making and/or increased risk taking. These links have not yet been investigated in the domain of sports and exercise. However, poor decisions or excessive risk behavior would be highly detrimental to sporting performance and, in some cases, even cause physical harm. Here, we address this gap by assessing if boredom proneness is linked to general risk taking, sport-specific risk taking, and to regrets about sports-specific decision errors with respect to acting too risky or too passively. **Methods**: *N* = 936 athletes (27.6 ± 9.0 years, 89.6% men): *n* = 330 Climbers (31.8 ± 10.7 years, 82.4% men), *n* = 83 Snowboarders (29.9 ± 8.3 years, 79.5% men), and *n* = 523 Esports athletes (24.6 ± 6.3 years, 95.8% men) completed the Short Boredom Proneness Scale (SBPS), along with measures for objective risk taking (Balloon Analogue Risk Task; BART), subjective risk taking (general willingness to take risks), as well as assessments for sport-specific risk taking and regrets for sports-specific decision errors (taking too many risks, failing to act at all). In the two extreme sports samples (i.e., climbers and snowboarders), we additionally assessed self-selected outcome certainty in a hypothetical sports-specific scenario where an error would result in physical harm. **Results**: A series of multiple regression analyses revealed that boredom proneness was unrelated to objective and subjective general risk taking, but a significant predictor of sport-specific risk taking and higher risk taking in the sports scenario (climbers and snowboarders only). Most importantly, boredom proneness predicted regrets for taking too many risks and being too passive. Exploratory post-hoc analyses further indicated that boredom proneness in extreme sports athletes was lower than in esports athletes. Higher boredom proneness was significantly related to lower skill levels across all kinds of sport. **Discussion**: Across three athlete samples, boredom proneness was unrelated to general risk taking but significantly related to poorer decision making, as indicated by regrets about acting too risky and too passively, as well as demanding a significantly lower safety threshold to make a risky sports-specific choice. While at odds with the often-reported link between boredom proneness and risk taking, these results are consistent with the conceptualization of boredom proneness as a maladaptive self-regulatory disposition that leads to noisy decision making in sports. In addition, we provide preliminary evidence that boredom proneness covaries with self-selection into specific types of sports and might also stand in the way of skill acquisition in sports.

## 1. Boredom Proneness Predicts Self-Assessed Decision Errors in Sports but Is Unrelated to Risk Taking in General

Interest in boredom research has surged in recent years [1,2,3]. A substantial body of theoretical and empirical work indicates that the experience of boredom affects goal-directed behavior in a wide range of settings and situations [4,5,6]. Interestingly, boredom’s potential impact on sports performance and behavior has been acknowledged as early as 1926 [7]. However, research that directly investigates boredom in sports is still scarce, and this has only recently started to change [8]. For example, one study has shown that high *boredom proneness*—the relatively stable disposition to be frequently and intensely bored—is linked to less physical exercise in the general population [9]. In addition, episodes of boredom have been found to impair performance in a sample of elite and sub-elite athletes [10]. While these initial results support the assumption that boredom matters for sports performance and behavior, the link between them is still poorly understood.

To better understand the potential role of boredom in sports, it is instructive to focus on its function for human behavior in general [11,12,13,14]. In a nutshell, recent functional accounts on boredom—defined as the aversive experience that arises when an individual wants to engage in more satisfying activities but fails to do so [15]—argue that boredom operates as a signal to signify that whatever one is currently doing is not satisfying enough and one should rather attend to something else [16]. Importantly, as a transient state, boredom is a signal that is neither inherently good nor bad. Boredom merely signifies the need to change *something*; the signal itself does not specify *what* this something is. However, this is not necessarily the case for individual differences in boredom, as a large body of research has rather unanimously linked boredom proneness with adverse outcomes [17]. Indeed, high boredom proneness has been linked to failures to effectively self-regulate in various contexts [18,19,20,21], thereby solidifying boredom’s role as a critical sensation in human self-regulation [16]. As self-regulation is of critical importance in sports [22], boredom proneness might also covary with how athletes manage the self-regulatory demands of their sports, as well as the type of sport they select themselves into in the first place.

One way in which boredom proneness might impair effective self-regulation is via its effect on decision making. Boredom has been found to increase reward sensitivity [23], thereby biasing decisions towards more readily available rewards, which might reduce the efforts that are directed towards rewards that accrue in the long term (e.g., getting physically fit and healthy due to regular exercise or excelling at the elite level in one’s sport). In addition to affecting the frequency and quality of sports behavior, such a boredom-induced decision bias might manifest itself in behavioral choices that are deemed risky. For example, a snowboarder might attempt a very challenging jump that, if successfully landed, can all but guarantee success but could lead to a ruptured cruciate ligament when the athlete gets the landing wrong. Tentative support for this assumption comes from research outside the sports domain, where boredom proneness has been linked to engagement in various risk behaviors such as gambling [24], drug abuse [19], and flaunting of COVID-19 restrictions [18]. However, boredom proneness might not directly lead to more risk taking. Going beyond self-report measures of risk taking, Yakobi and Danckert (2021) found no relationship between an objective measure of risk taking and boredom proneness [25]. To explain this finding, the authors propose that boredom proneness may lead to noisy decision making that can appear risky at times but does not necessarily reflect risk preferences. Supporting this hypothesis, they showed that boredom-prone people tend to switch more between choices, irrespective of the risk attached to these choices [25]. Electrophysiological evidence further substantiated these behavioral findings for a reduced feedback sensitivity in boredom-prone people. This indicates that boredom proneness might render people less effective in basing their choices on the reward structure of the task at hand [25], therefore making self-regulatory decision failures more likely in boredom-prone individuals [26]. Indeed, boredom proneness is consistently linked with poor self-control [9,18,27] and has not only been associated with risk behavior but also with more general self-regulatory failures to launch [26] and disengage [28]. Therefore, we propose that boredom proneness predicts self-regulatory decision failures in sports, some of which should pertain to increased risk taking (e.g., attempting a dangerous move in one’s sport) whereas others should not (e.g., acting too passive in a critical situation in one’s sport). Investigating this proposition is highly relevant, as noisy decision making in sports might not only impair performance but could also be a source of harm to the self or others (for example by increasing the likelihood of sports injuries). However, although the domain of sports is ideally suited to investigate the relevance of boredom proneness with respect to self-regulatory decision failures, the importance of unpacking the role it plays extends well beyond the sporting domain.

To investigate this issue in a sports context, we turned to extreme sports (i.e., climbing and snowboarding) and esports athletes. Extreme sports athletes are a suitable population for testing our hypotheses, as incurring increased risks of physical harm is part of the definition of what makes a type of sport extreme [29]. Thus, per definition, the choices extreme sports athletes must make in their sports involve taking actual risks of physical injuries. For example, a rock climber attempting to set a new speed record might choose to try a potentially faster but more dangerous route. Esports athletes constitute another suitable population for testing our hypotheses, as esports athletes also exhibit (typically non-physical) risk taking in their sport regularly [30]. They consistently have to make split-second decisions that can drastically alter the outcome of the game and the competition. For example, an esports athlete specializing in the first-person shooter ‘Counter-Strike’ may attempt to surprise opponents with the decision to leave their cover, which exposes them to an increased risk of getting hit. Thus, if boredom proneness as a domain-general trait indeed increases risk taking, a positive correlation should emerge between boredom proneness and both objective and subjective measures of risk taking, as well as with sport-specific risk taking. If, on the other hand, boredom proneness rather affects self-regulatory decision making on a more general level by making choices noisier [25], no correlation with an objective measure of risk taking should emerge. Noisier (i.e., poorer) choices should instead manifest themselves in athletes’ regret about their sporting choices more generally. Thus, across two different types of sport, we expect boredom proneness to predict regret about taking too many risks but also regret about having been too passive. To this end, we collected data from two samples of extreme sports athletes and one sample of esports athletes who had similar training schedules and were engaged in sports that frequently required sports-related risk taking.

## 2. Method

Materials, data, and analysis scripts can be found at https://researchbox.org/538 (accessed on 10 March 2022). Participants in this study also constitute a subsample of a publication unrelated to the investigation of boredom proneness [31].

### 2.1. Design, Participants, and Sample Size Considerations

We collected data from three different groups of athletes (climbers, snowboarders, esports). We refrained from adding a non-athlete control group, as the present research focused on how the relevance of boredom proneness generalizes within the sporting domain but not to other domains. Each survey was programmed in Qualtrics and differed only in the sport-specific questions. We classified responses as started when participants gave their informed consent and as eligible for data analyses when they filled out the boredom proneness scale, the second-to-last questions in the entire survey. In total, 936 athletes completed the survey. Groups varied in size, from 83 snowboarders to 330 climbers to 523 gamers. However, the collected sample sizes allow for a reliable test (80% power) of *r* ≥ 0.297 in case of the smallest group (snowboarders) and down to *r* ≥ 0.122 and *r* ≥ 0.091 in case of the largest group (esports athletes) and all participants, respectively [26].

We recruited gamers, climbers, and snowboarders via various online forums, climbing gyms, and (inter)national climbing and snowboarding federations. Table 1 gives an overview of the participant flow and the basic demographics as well as training schedules of each discipline.

### 2.2. Procedure

Participants chose between a German and an English version of the survey, gave their informed consent, provided demographic information (e.g., age and gender), and indicated their experience, training routines, and participation in competitions. Participants then performed the Balloon Analogue Risk Task (BART [32]) as a measure of general risk taking and answered sports-specific questions: They indicated their general willingness to take sport-specific risks [33] and responded to questions that directly pertained to ineffective self-regulatory decisions (i.e., either taking too many risks or acting too passively) in their respective sports. At the end of the survey, participants filled out the short boredom proneness scale [34], indicated whether they completed the survey carefully (98% reported doing so across all completions), and were debriefed. The procedure followed the guidelines laid out in the Declaration of Helsinki (1975). Participation was voluntary and happened only after participants had given prior informed consent. The present study falls outside the range of research requiring a dedicated IRB statement according to the position of the ethics committee of the authors institution.

### 2.3. Material

#### 2.3.1. Boredom Proneness

All three groups completed the short boredom proneness scale (validated English version [34], validated German version [35]), an eight-item questionnaire assessing trait boredom proneness (Cronbach’s α = 0.84) on a 7-point Likert scale.

#### 2.3.2. Risk-Taking Behavior: Balloon Analogue Risk Task (BART)

Participants performed 20 trials of the Balloon Analogue Risk Task (BART [32]), programmed in Javascript. In each trial of the BART, participants have two options. They can pump up a balloon with a mouse click and increase the balloon’s value (in hypothetical points) with each pump, or they can decide to stop pumping and save its value to a temporary bank. Pumping too much, however, can lead to the bursting of the balloon, eradicating its current value. The number of bursts was used as a proxy for general risk taking. The number of pumps required to let the balloon burst was predetermined in each trial, and the order of these breaking points was held constant across participants. Breaking points ranged from 5 to 59 (*M* = 32), ensuring that even high levels of risk taking would require a manageable number of clicks.

#### 2.3.3. Self-Reported Risk-Taking Behavior and Decision Making

**General Willingness to Take Risks.** We asked all participants to rate their willingness to take risks in general on one widely used and validated single item that uses a 7-point response scale [33,36].

**Sports-Specific Questions.** To assess noisy decision-making regarding sports-related decisions, athletes indicated their immediate and delayed regret after taking a risk or being too passive in their sport. Furthermore, we asked them whether others would describe their sport-specific style as risky.

Four questions were devised to assess these variables: Athletes indicated their agreement with the statements “I get mad at myself when I took a risk *while climbing* [*in a game*/*while snowboarding*]” and “Others would describe my *climbing* [*play*/*riding*] style as risky” on 6-point scales ranging from *I totally disagree* to *I totally agree*. After that, they were asked to assess themselves on the following items: “I often get mad at myself after I took a risk *while climbing* [*in a game*/*while snowboarding*],” which was accompanied by examples (e.g., climbing: skipping a hold, gaming: overextending into enemy territory, snowboarding: dangerous overtaking maneuver) and “I often get mad at myself that I *climbed* [*played*/*rode*] too passively,” both to be answered on a 100-point visual analog scale ranging from *never* to *always*. All sport-specific questions focused on distinct contents and were therefore analyzed as single items in the analyses reported below. Therefore, no measures of internal consistency can be provided for these questions.

***Risk Scenario.*** Extreme sport athletes make choices that carry the risk of actual physical harm. To assess the outcome certainty they required to incur such risks, we developed hypothetical sport-specific scenarios together with experts in the respective sport (an experienced athlete and a coach). Here, participants should imagine that they climb (ride a jump) at their maximum capacity and can skip a hold (try a new trick). This would either allow them to win time and save energy (increase experience and trust) or result in a loss of time and energy (being blocked for the next time). Crucially, failing could also result in injury. Participants then responded to the question “How certain would you have to be that skipping the hold will be successful/you will land the trick” on a 7-point scale, ranging from *I am certain that it is not successful* to *50/50* (midpoint) to *I am certain that it is successful*, and *I wouldn’t have to be certain that I land the trick* to *50/50* (midpoint) to *I would have to be certain that I will land the trick*, respectively. Therefore, lower scores represent a higher willingness to take risks as athletes require less outcome certainty to take the risky action (i.e., skipping a hold or trying a new trick).

## 3. Results

### 3.1. Boredom Proneness and Risk Taking

While controlling for differences between the three groups of athletes (in order to assess if results are robust across different sports types), regressing self-reported risk taking on boredom proneness rendered no significant effects, *F*(3, 932) = 1.72, *p* = 0.162, *R*^2^ = 0.006, with no differences between the three groups of athletes, all *p*s ≥ 0.070. Similarly, boredom proneness was no significant predictor for risk-taking behavior in the BART when we looked at the average number of pumps, β = 0.017, *t*(929) = 0.49, *p* = 0.621, or the number of bursts, β = 0.026, *t*(932) = 0.78, *p* = 0.434. For risk-taking behavior in the BART, sports-related differences existed, as indicated by the significant dummy-coded predictors for climbing (vs. esports), βs ≥ 0.209, *t*s ≥ 6.22, *p*s < 0.001, and snowboarding (vs. esports), βs ≥ 0.088, *t*s ≥ 2.56, *p*s ≤ 0.011, leading to overall significant regression models, *F*s ≥ 13.43, *p*s ≤ 0.001, *R*^2^s ≥ 0.042. Taken together, extreme sports athletes took more risks in the BART than esports athletes (as we have reported elsewhere [31]). Still, boredom proneness predicted neither self-reported nor objective risk taking.

### 3.2. Boredom Proneness and Sports-Specific Decision-Making

Next, we regressed the self-report of whether others would describe one’s style as risky on boredom proneness while controlling for differences between the three groups of athletes. This resulted in an overall significant regression model, *F*(3, 931), = 26.11, *p* < 0.001, *R*^2^ = 0.078, with boredom proneness being a significant predictor, β = 0.121, *t*(931) = 3.69, *p* < 0.001. Once again, group differences emerged, as indicated by the significant dummy-coded predictor for climbing (vs. esports), β = −0.236, *t*(931) = 7.18, *p* < 0.001, but not snowboarding (vs. esports), β = 0.008, *t*(931) = 0.23, *p* = 0.819. Similarly, need for safety in the risk scenario (only assessed for extreme sports athletes) was related to boredom proneness, β = −0.134, *t*(409) = 2.64, *p* = 0.009, leading to an overall significant regression model, *F*(2, 409) = 3.48, *p* = 0.032, *R*^2^ = 0.017. Both results indicate that higher boredom proneness is associated with higher sports-specific risk taking in extreme sports and esports alike.

Focusing on the potential consequences of noisy decision-making in sports, we then analyzed how much variance in immediate and delayed regret after taking a risk and being too passive can be explained by our background variables (sport, age, and gender), general risk taking, and boredom proneness. Therefore, we ran three regression analyses using a hierarchical procedure. First, we controlled for sport, age, and gender. Then, the number of bursts in the BART was added as a proxy for risk-taking behavior. Finally, the independent variable of interest, boredom proneness, was added to investigate its potential explanatory power beyond our control variables and an objective measure of general risk taking.

Table 2 provides an overview of the results of these analyses and bivariate correlations between all predictor variables along with boredom proneness are depicted in Table 3. Boredom proneness was significantly related to all but one variable after controlling for the set of control variables and risk taking. Immediate and delayed regret after taking a risk were negatively associated with risk taking but positively related to boredom proneness (for immediate regret, this relationship was not significant). In contrast, regret about having been too passive was positively related to boredom proneness but not to risk taking.

### 3.3. Exploratory Analyses: Boredom as Dependent Variable

**Figure 1:**
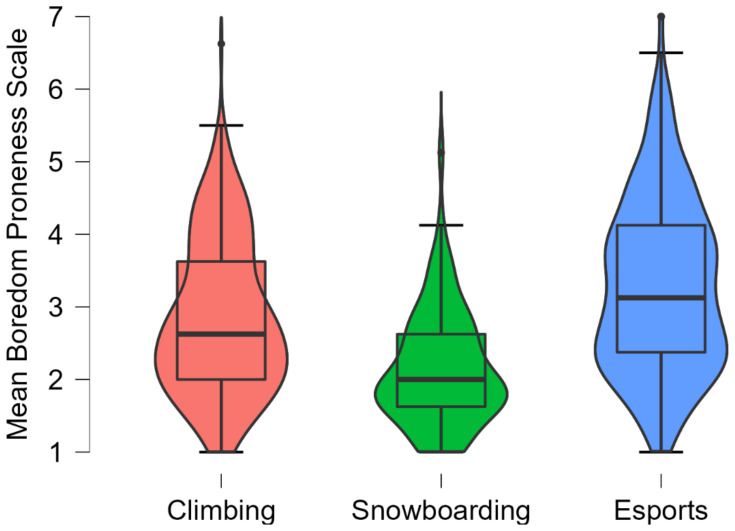
Violin plots that represent sample distributions, as well as the differences in boredom proneness between climbers, snowboarders, and esports athletes.

Next, we tested whether boredom proneness differed between the three groups of athletes. This was the case, *F*(2, 262.4) = 64.77, *p* < 0.001, η^2^ = 0.086, 95%-CI = [0.054; 0.120]. Bonferroni-corrected follow-up comparisons revealed significant differences between all three groups, all *p*s < 0.001. More specifically, esports athletes exhibited the largest levels of boredom proneness (*M* = 3.29, 95%-CI = [3.18; 3.39], *SD* = 1.20), followed by climbers (*M* = 2.84, 95%-CI = [2.73; 2.96], *SD* = 1.06) and snowboarders, *M* = 2.15, 95%-CI = [1.98; 2.32], *SD* = 0.79 (see Figure 1). The differences also hold when controlling for differences in age and gender in a multiple linear regression, *p*s ≤ 0.049.

Lastly, simultaneously regressing boredom on age, gender, self-reported skill, and years of experience while controlling for differences between sports revealed significant effects of gender, β = −0.136, *t*(914) = 4.30, *p* < 0.001, age, β = −0.191, *t*(914) = 4.78, *p* < 0.001, and self-reported skill (z-scored), β = −0.069, *t*(914) = 2.13, *p* = 0.033, on boredom proneness, *F*(6, 914) = 25.10, *p* < 0.001, *R*^2^ = 0.141. Experience was not a significant predictor of boredom proneness, β = −0.032, *t*(914) = 0.76, *p* = 0.447. Thus, being male, being younger, and being less skilled were all linked to higher boredom proneness.

## 4. Discussion

The potential relevance of boredom in sports has long been overlooked by researchers [8]. Here, we address this gap by investigating the link between boredom proneness and risk taking in sports, as prior research has frequently linked boredom to risky behavior [24,37].

### 4.1. Boredom Proneness Is Associated with Noise, Not with Risk

Interestingly, we observed no relationship between boredom proneness and general risk taking. This was true for objective (the Balloon Analogue Risk Task; BART) and subjective measures of risk taking. In addition, this pattern held across three different athlete samples that differed with respect to the actual risks for physical harm their respective sports imposed (high risk, snowboarders and climbers; low risk, esports athletes). These findings speak against a link between boredom proneness and general risk taking among athletes and exercisers. However, they are consistent with recent experimental research outside the sports setting, proposing that boredom proneness does not increase risk taking per se but instead leads to poorer decision making [25] which *can* lead to riskier behavior but can also lead to failures to act at all [26,28]. In line with this assumption, boredom-prone athletes (across samples) stated that others would also rate their style as being risky. Consistent with this, boredom-prone athletes from two extreme sports samples demanded less outcome security in a sports-specific risk scenario, where potential success (e.g., winning) was pitted against potential risks (e.g., getting injured). Supporting the claim that this does not reflect an increased risk appetite but rather noisier decision making, boredom-prone athletes reported to frequently experience regrets about taking too many risks in their sports. Thus, they reportedly acted riskier and tended to regret these risks afterwards. Supporting the idea that boredom proneness does not merely bias decisions towards greater risk taking, boredom-prone athletes also stated to experience regrets for having been too passive. Thus, boredom-prone athletes not only regret the risks they take but also the actions they fail to take. This is in line with the understanding of boredom proneness as a disposition linked to poor self-regulation in general [18,26]. Attesting to the robustness of these findings, effects were robust even when controlling for demographic variables, as well as for objective and subjective indicators of general risk taking.

### 4.2. Boredom Proneness Is Associated with Athletic Level and Sporting Choice

Results from exploratory post-hoc analyses suggest that, in addition to covarying with self-regulatory choices in sports, boredom proneness might also play a role concerning the skill level athletes achieve in their sports. Across samples, regression analyses revealed that skill level was a negative predictor of boredom proneness: Those who had achieved high skill levels in their respective sports tended to be less boredom prone. In light of the crucial importance of self-regulation for sporting success [22] and the known negative relationship between boredom proneness and (measures of) self-regulation [38], one interpretation of this finding is that boredom proneness might potentially stand in the way of achieving sporting excellence.

In further exploratory post-hoc analyses, we found that boredom proneness differed between samples. Esports athletes were significantly more boredom prone than extreme sports athletes. One tentative explanation for this finding is that boredom proneness might affect in which sports aspiring athletes self-select themselves into. Thus, based on the findings present here, boredom prone people might be more drawn to take up esports and less inclined to take up extreme sports. Importantly, although these results were robust when various demographic variables were controlled for, it should be noted that these findings stem from exploratory post-hoc analyses that warrant further research. Ideally, this research should rely on longitudinal studies to estimate the temporal dimension of these relationships (e.g., whether higher levels of boredom proneness precede lower skill levels and self-selection into sports).

Lastly, boredom proneness varied as a function of age and gender in ways that are consistent with the extant literature [2] . Boredom proneness was negatively related to age, and males were more boredom prone than females. Results of these exploratory analyses have practical implications that are potentially highly relevant. For example, if boredom proneness covaries with sporting choices and if boredom proneness varies as a function of demographic variables, then boredom proneness could be a psychological variable for practitioners to consider when tailoring sporting programs.

### 4.3. Further Considerations and Directions for Future Research

That some people take more risks or are drawn to riskier sports than others might also be interpreted in terms of a desire to satisfy a higher need for sensation seeking—or the “desire for novel and intense stimulation” [39] (p. 342). Indeed, sensation seeking has been linked to various risk behaviors inside and outside the sporting domain [40]. Importantly, self-report measures of sensation seeking include a boredom susceptibility subscale, suggesting that boredom is subsumed within a broader sensation seeking construct [41]. However, this does not reflect the scientific definition of boredom (as a state of wanting to engage with something but failing to do so), and it is also at odds with empirical findings that report only modest relationships between boredom proneness and sensation seeking, as well as showing that boredom proneness and sensation seeking independently contribute to the prediction of various outcomes [39]. Thus, while sensation seeking is by definition linked to the search for stimulating and novel experiences, boredom does not necessarily imply such a directed search, and boredom proneness has even been characterized as a self-regulatory “failure to launch” [26] (p. 1), emphasizing the fact that one regularly finds oneself in unsatisfying states. Our findings are more in line with this latter conceptualization of boredom proneness, as we did not observe a link between boredom proneness and risk taking in the BART, which has been found to be related to sensation seeking in past studies (e.g., [32]). In addition, our results are in line with recent work indicating that boredom proneness impairs self-regulatory choices by adding noise [25]: Although high boredom proneness was linked to increased other-ascribed risk taking in the athletes’ sport of choice, it was also linked to being too passive in critical situations, and in general to a lower need for outcome safety when making choices in sports. Thus, while sensation seeking potentially affects self-selection into specific types of sport, and behavior in those sports, via an increased risk appetite [42,43], the present research on boredom points to a decidedly different role for boredom proneness: boredom proneness has been linked to failures to effectively self-regulate, and in sports, such failures might manifest themselves in a many ways (e.g., by impairing performance, participation, or focus), only one of which might be increased involvement in risky behaviors. Thus, while our findings are at odds with an explanation through the lens of sensation seeking, we did not directly measure sensation seeking. In turn, further research should directly assess the joint and the differential impact of boredom proneness and sensation seeking on sporting choices.

Another promising direction for further research pertains to the conceptualization of boredom proneness in general and its differentiation from state boredom (interestingly, recent work on state boredom has rather consistently shown that being bored increases risky behavioral choices [37,44]). According to recent empirical and theoretical work, state boredom is a functional signal that signifies the need to change something [11,13,16]. Crucially, this signal is understood as being an impartial signal for change that could give rise to adaptive and maladaptive behaviors alike. However, this idea is not incorporated in the concept of boredom proneness, which is based on questionnaires developed at a time when no generally accepted definition and theory of boredom was available [45]. Therefore, research on trait boredom has been conducted largely independently from conceptual and empirical work on state boredom [11]. As a consequence, boredom proneness captures how often and how intensely people are bored and whether they perceive their life as boring in general [17] but is agnostic to whether an individual experiences the need to change something about the current situation [46]. Consequently, boredom proneness strongly overlaps with other constructs such as self-control [9,28,38], which might explain why it is often associated with risk taking. Developing measures of trait boredom that better incorporate core elements of the definition of boredom will likely show a more nuanced picture of the relationship between boredom, risk taking, and decision making in sports and exercise.

## 5. Conclusions

While athletes’ boredom proneness seems unrelated to risk taking in general, boredom-prone athletes take more sports-specific risks. They require less outcome security for their choices in ambiguous sporting scenarios. Supporting the idea that boredom proneness adds noise to self-regulatory choices (i.e., essentially makes choices poorer), boredom-prone athletes regret acting too risky and being too passive in their sports. These results suggest that boredom proneness plays a critical role with respect to self-regulatory choices in sports. In addition, boredom proneness was related to significantly lower sport-specific skill levels, and athletes from esports were more boredom prone than extreme sports athletes. Thus, the present research substantiates the notion that boredom matters in the sports and exercise context and that more research on boredom in this domain is dearly needed [8].

## Figures and Tables

**Table 1:** Participant flow, basic demographics, experience, training schedule, and skill as a function of group.

Variable	Climbers	Snowboarders	Esports Athletes	Total
*N* survey starts	570	120	1288	1978
*N* (% completions)	330 (58%)	83 (69%)	523 (41%)	936 (47%)
Age	18–67 years old (*M* = 31.8 *SD* = 10.7)	18–62 years old (*M* = 29.9 *SD* = 8.3)	18–66 years old (*M* = 24.6 *SD* = 6.3)	*M* = 27.6 *SD* = 9.0
Gender	82% male, 18% female	80% male, 20% female	95% male, 3% female, 2% other	89% male, 10% female, 1% other
Experience [years]	*M* = 10.1 *SD* = 9.2	*M* = 15.3 *SD* = 7.7	*M* = 5.3 *SD* = 5.1 ^a^	*M* = 7.9 *SD* = 7.8
Training Sessions [per week]	*M* = 2.7 *SD* = 1.5	*M* = 1.9 *SD* = 2.7	*M* = 4.0 *SD* = 2.7 ^a^	*M* = 3.4 *SD* = 2.5
Length Training Session [minutes]	*M* = 118.1 *SD* = 61.9	*M* = 84.3 *SD* = 98.0	*M* = 111.2 *SD* = 76.5 ^a^	*M* = 111.4 *SD* = 74.2
Self-Reported Skill	*M* = 8.2 *SD* = 1.2 ^b^	5% Beginner 37% Advanced 35% Skilled 23% Expert	*M* = 5.2 *SD* = 1.6 ^a,c^	
Highest Contest Participation	91% none or amateur 4% regional 4% national 1% international	87% none or amateur 4% regional 4% national 6% international	65% none or amateur 16% regional 7% national 13% international ^a^	76% none or amateur 11% regional 5% national 8% international

*Note.* ^a^ Esports athletes could decide on one of 71 popular multiplayer online games or list their own choice as the main game of interest and gave responses regarding playing this game (series). ^b^ Climbers indicated their skill on the 12-point Union Internationale des Associations d’Alpinisme-Scale. ^c^ Esports athletes indicated their skill on a 9-point scale ranging from *Beginner* to *Master.*

**Table 2:** Linear hierarchical regression models regressing sports-related risk taking on a set of control variables (group, gender, and age), risk taking, and boredom proneness.

Dependent Variable	β (Δ*R*²)		
	Control Variables	*Risk Taking*	*Boredom Proneness*
Immediate regret after taking a risk (1–6 scale)	(0.069) ***	−0.120 (0.013) ***	0.060 (0.003)
Delayed regret after taking a risk (0–100 scale)	(0.089) ***	−0.100 (0.009) **	0.067 (0.004) *
Regret about having been too passive (0–100 scale)	(0.046) ***	0.017 (0.000)	0.147 (0.019) ***

*Note.* Higher values express stronger agreement. The β-coefficients stem from the last model that includes all variables. *** *p* < 0.001, ** *p* < 0.010, * *p* < 0.050.

**Table 3:** (Non)Parametric correlations between gender, age, skill (self-reported), highest competition level, experience, and boredom proneness across all sports.

Variable	(1)	(2)	(3)	(4)	(5)	(6)
(1) Gender	*n* = 926	*r* = 0.057, *p* = 0.085	*r* = −0.096, *p* = 0.003	*ρ* = −0.077, *p* = 0.019	*r* = 0.054, *p* = 0.098	*r* = −0.178, *p* < 0.001
(2) Age		*n* = 930	*r* = −0.006, *p* = 0.865	*ρ* = −0.133, *p* < 0.001	*r* = 0.585, *p* < 0.001	*r* = −0.255, *p* < 0.001
(3) Skill (z-scored)			*n* = 936	*ρ* = 0.320, *p* < 0.001	*r* = 0.324, *p* < 0.001	*r* = −0.061, *p* = 0.062
(4) Competition level				*n* = 936	*ρ* = 0.064, *p* = 0.050	*ρ* = 0.041, *p* = 0.205
(5) Experience (in years)					*n* = 936	*r* = −0.242, *p* < 0.001
(6) Boredom Proneness						*n* = 936

*Note.* Correlations between the ordinal-scaled competition level and all other variables are nonparametric Spearman *ρ* coefficients; all other correlations are Pearson correlations (*r*). Reasons for varying sample sizes were that (a) for correlations with gender, only *male* (0) and *female* (1) participants were included in the analyses, and that (b) not all participants indicated their age. Years of experience correlated with boredom proneness in (almost) the same way that age did. Partialing out the apparent confound age due to the correlation between age and years of experience still rendered a significant correlation between years of experience and boredom proneness, *r*(927) = −0.117, *p* < 0.001, albeit significantly weaker, z = 3.94, *p* < 0.001.

## Data Availability

Data and materials are available at https://researchbox.org/538 (accessed on 10 March 2022).
